# Longitudinal association between frailty and pain in three prospective cohorts of older population

**DOI:** 10.1016/j.jnha.2025.100537

**Published:** 2025-03-23

**Authors:** Hongcheng Huang, Linghao Ni, Lyuhan Zhang, Jiawei Zhou, Bin Peng

**Affiliations:** College of Public Health, Chongqing Medical University, Chongqing, 401331, China

**Keywords:** Frailty, Pain, Longitudinal association, Epidemiology

## Abstract

**Background and objectives:**

As the global population ages, frailty and pain have become two significant health issues that impact the quality of life in older adults. Previous studies have not thoroughly explored the relationship between them. This study aims to investigate the longitudinal association between frailty and pain using data from prospective cohorts in China (CHARLS), the United Kingdom (ELSA), and the United States (HRS).

**Methods:**

This study utilized data from three prospective cohort studies: the China Health and Retirement Longitudinal Study (CHARLS), the English Longitudinal Study of Ageing (ELSA), and the Health and Retirement Study (HRS). Frailty status was assessed using the Rockwood frailty index and categorized into robust, pre-frail, and frail. Pain was evaluated by self-reports. Pain degrees were categorized into mild, moderate and severe. Pain areas were grouped into four main areas: head and neck, trunk, limbs, oral. Generalized linear mixed-effects models were employed to analyze the longitudinal relationship between frailty and pain while adjusting for covariates, including gender, age, marital status, education level, sleep quality, smoking, drinking, hypertension, and diabetes.

**Results:**

According to the inclusion and exclusion criteria, 10,624 participants from CHARLS (47% female, mean age: 60.76 years), 4945 participants from ELSA (52.2% female, mean age: 70.05 years), and 11,439 participants from HRS (55.8% female, mean age: 69.28 years) were included in the subsequent analysis. Compared to robust individuals, those in pre-frail and frail states showed a significantly increased risk of experiencing pain. In all three cohorts, pre-frail individuals had a 3.82-fold increased likelihood of pain compared to robust individuals (OR = 3.82, 95%CI = 3.51–4.15, p-value < 0.001, CHARLS), 4.29-fold (OR = 4.29, 95%CI = 3.74–4.93, p-value < 0.001, ELSA), and 4.17-fold (OR = 4.17, 95%CI = 3.81–4.57 p-value < 0.001, HRS). Frail individuals had a 10.44-fold increased likelihood of pain (OR = 10.44, 95%CI = 9.05–12.04, p-value < 0.001, CHARLS), 10.14-fold (OR = 10.14, 95%CI = 8.05–12.76, p-value < 0.001, ELSA), and 13.27-fold (OR = 13.27, 95%CI = 11.71–15.03, p-value < 0.001, HRS).

**Conclusion:**

This study demonstrates that frailty significantly impacts the risk of pain, the degree of pain, and the areas of pain. And this association is consistently observed across older populations in different countries. Future pain management strategies should incorporate frailty assessments to mitigate the adverse effects of pain on the health of older adults.

## 1 Introduction

Pain is “an unpleasant sensory and emotional experience associated with actual or potential tissue damage or described in terms of such damage” [1]. While everyone experiences pain at some point, older adults are generally at higher risk of pain [2]. Studies have shown that the prevalence of pain in older adults ranges from 24% to 72% [3]. Research on individuals aged 80 or older found a pain prevalence rate of 50.4% to 63% [4,5]. Pain significantly impacts individuals and society [6]; it is recognized by the World Health Organization and the United Nations as a global issue and is increasingly considered an independent disease [7]. In the United States, pain is one of the most prevalent and costly medical issues, affecting an estimated 100 million Americans, which surpasses the estimated impact of cancer, heart disease, and diabetes [8,9]. Additionally, pain can lead to future disability, reduced mobility, institutionalization, and increased mortality [10,11], severely impacting individuals’ quality of life and normal aging [12]. Review studies have also indicated that pain is a risk factor for suicidal behavior in the older adult [13]. These studies underscore the need for a comprehensive approach to timely pain prevention.

Studies investigating pain determinants in various populations have shown that age, female gender, low socioeconomic status, and lower education levels are important risk factors for experiencing pain. Comorbidities, cognitive impairment, and depression are also associated with pain, while physical activity reduces the likelihood of pain [14]. Frailty is a clinical condition characterized by reduced physiological reserves and increased vulnerability to stressors [15]. Recent studies indicate that frailty is closely related to pain in older adults [16]. Moreover, research suggests a bidirectional relationship between pain and frailty: pain predicts the development of frailty, while frailty predicts the development or worsening of pain [17].

Frailty is a dynamic process that can gradually improve or worsen, with its prevalence increasing with age [18]. Individual frailty can significantly lead to various adverse outcomes, including increased risks of disability, hospitalization, premature mortality, and impaired resilience to stressors [19]. Debate continues regarding the most suitable operational definitions and assessment tools for defining frailty in clinical practice. The frailty phenotype described by Fried is based on five criteria associated with impaired physical capacity [20], while the frailty index (FI) proposed by Rockwood and Mitnitski, which is based on the accumulation of age-related deficits, is the most commonly used [21]. In principle, the FI is calculated by determining the proportion of health deficits (which includes symptoms, signs, diseases, disabilities, or abnormalities from laboratory tests, radiological exams, or electrocardiograms) relative to the total number of considered deficits. A higher FI indicates more health deficits, corresponding to increased frailty. Therefore, using the FI for regular frailty assessment is crucial for preventing or delaying pain and its associated negative impacts.

Despite the well-documented global impact of pain and its high prevalence among older adults, studies on the relationship between frailty and pain are limited. Most studies exploring the relationship between pain and frailty are cross-sectional or systematic reviews [22]. Existing longitudinal studies are also mostly restricted to a particular region or population [23,24]. Therefore, this study examined the longitudinal associations between frailty and the occurrence of pain, the degrees of pain, and the areas of pain, using data from three longitudinal cohorts: the Chinese Longitudinal Study of Health and Retirement (CHARLS), the English Longitudinal Study of Aging (ELSA), and the US Health and Retirement Study (HRS). Our study offers new insights into the relationship between aging and health in large population groups, forming an important basis for interventions and policies specifically designed for this crucial yet under-researched population in the future.

## 2 Methods

### 2.1 Study design and participants

CHARLS, ELSA, and HRS are prospective, nationally representative cohort studies conducted in China, the United Kingdom, and the United States, respectively [[25], [26], [27]]. In this study, Wave 1 of CHARLS (2011), Wave 6 of ELSA (2012), and Wave 11 of HRS (2012) were used as baseline data. Each subsequent follow-up survey (CHARLS: 2013, 2015, 2018, 2020; ELSA: 2014, 2016, 2018, 2021–2023; HRS: 2014, 2016, 2018, 2020) was included until the latest follow-up. Ethical approval was obtained from the Institutional Review Boards of Peking University for CHARLS, the London Multi-Centre Research Ethics Committee for ELSA, and the University of Michigan for HRS, with informed consent obtained from each participant in the three cohorts.

**Fig. 1:**
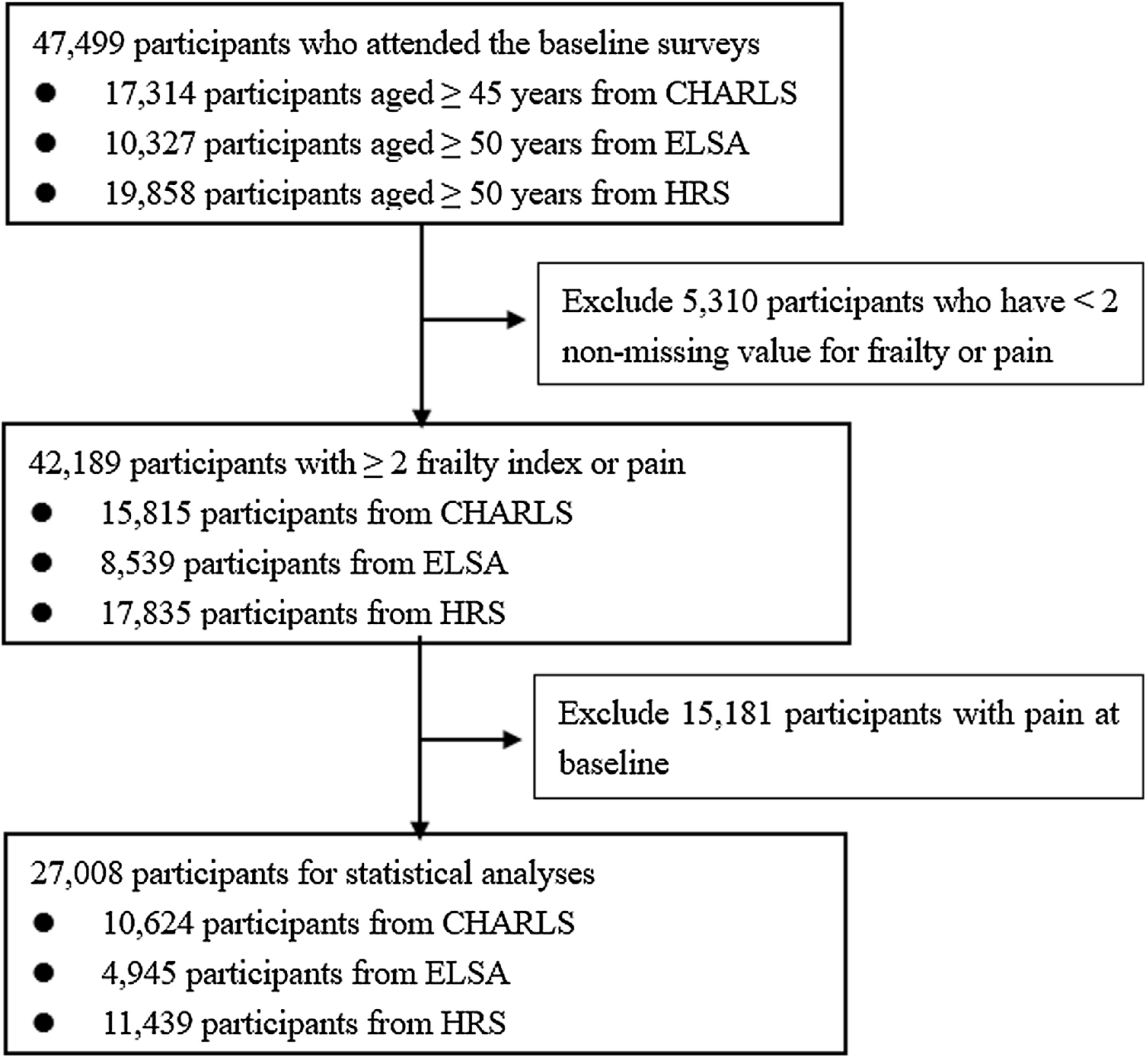
Selection process of the study population. CHARLS, China Health and Retirement Longitudinal Study; ELSA, English Longitudinal Study of Ageing; HRS, Health and Retirement Study; Fl, frailty index.

The exclusion criteria were the same for all three databases, with 2 main points: (1) having at least two non-missing values for frailty or pain; (2) having pain at baseline. Fig. 1 illustrates the selection process of the study population. Out of 47,499 participants from CHARLS, ELSA, and HRS, we excluded 5310 participants who have <2 non-missing values for frailty or pain during follow-ups. Additionally, 15,181 participants reporting pain at baseline were excluded. Ultimately, 27,008 eligible participants were included in the final analysis.

### 2.2 Pain assessment

Pain was primarily assessed by asking participants “Are you often troubled with any body pains?” or “Are you often troubled with pain?” Respondents answered “Yes” or “No,” allowing us to determine whether they were experiencing pain. Pain degrees were assessed by asking participants “How bad is the pain most of the time?” Respondents answered “Mild”, “Moderate” or “Severe,” allowing us to categorize pain degrees into three categories. Pain areas were defined based on 16 specific pain sites, then grouped into four main areas: head and neck, trunk (in chest, stomach, back, waist or buttocks), limbs (in shoulders, arms, wrists, fingers, legs, knees, ankles, or toes), oral (mouth or teeth).

### 2.3 Frailty assessment

In this study, the FI was constructed according to established standards described in previous research [21,28], based on multiple age-related health deficits. Frailty was assessed by calculating the FI score, which included 32 items from CHARLS, ELSA, and HRS data related to diseases, disabilities, physiological functions, self-rated health, depression, and cognition (see Supplementary Table S1 for details). Items 1–31 were classified as 1 (deficit present) or 0 (no deficit) according to the relevant cutoff values. Item 32 (cognitive score) was a continuous variable ranging from 0 to 1, where a higher cognitive score indicated lower cognitive ability. For each participant, FI was calculated as the sum of current health deficits divided by 32, resulting in a continuous FI ranging from 0 to 1. A higher FI indicates a higher degree of frailty. As suggested by previous studies [[28], [29], [30]], frailty status was classified into three categories: robust (FI ≤ 0.10), pre-frail (0.10 < FI < 0.25), and frail (FI ≥ 0.25).

### 2.4 Covariates

Covariates included gender, age, marital status, education level, smoking status, alcohol consumption, self-rated sleep quality, hypertension, and diabetes. To ensure consistency across CHARLS, ELSA, and HRS, marital status was classified into two categories: married or partnered and unmarried (separated, divorced, never married, or widowed). Education was divided into three levels: below high school, high school, and above high school. Smoking status was classified as never smokers and smokers, with smokers including both past and current smokers. Similarly, alcohol consumption was categorized as never drinkers and drinkers. Sleep quality was classified into two levels (good or poor) based on responses to the question “Sleep was restless during the past week? /How often do you feel really rested when you wake up in the morning?”

### 2.5 Statistical analysis

Continuous variables were presented as mean [standard deviation (SD)] or median [interquartile range (IQR)] and categorical variables were expressed as frequency (percentage). Baseline comparisons across different frailty statuses were performed using analysis of variance (ANOVA) or chi-square tests.

The longitudinal association between frailty and pain from baseline to the end of follow-up was analyzed using generalized linear mixed-effects models (GLMM) fitted through the R package “lme4”. Frailty status/frailty index was treated as a fixed effect, and time (follow-up year) and ID (the unique identifier for each participant) were treated as random effects. Using robust participants as the reference, eight GLMMs (four for frailty status and four for the frailty index) were fitted to each of the three study populations. Model 1 was unadjusted; Model 2 adjusted for gender, age, marital status, and education level; Model 3 further adjusted for smoking status, drinking status, and sleep quality based on Model 2; and Model 4 further adjusted for hypertension and diabetes based on Model 3.

In addition, we analyzed the association between frailty and pain areas using the same method described above based on the data from CHARLS and ELSA. And the association between frailty and pain degrees was analyzed by fitting Cumulative Link Mixed Models (CLMMs) using the “ordinal” R package based on the data from ELSA and HRS. This model is applicable to ordered categorical response variables and is able to incorporate both fixed and random effects. Pain degrees were used as the outcome variable, with mild as the control, to analyze the effect of frailty on the occurrence of higher pain degrees.

We reanalyzed the association between frailty and pain using the COX model as the sensitivity analysis, details in Table 4.

All statistical analyses were conducted using R software (version 4.3.3). All hypothesis tests were two-sided, with *P* < 0.05 considered statistically significant.

## 3 Results

### 3.1 Baseline characteristics of the study population

According to the inclusion and exclusion criteria, 10,624 participants from CHARLS (47% female, mean age: 60.76 years), 4945 participants from ELSA (52.2% female, mean age: 70.05 years), and 11,439 participants from HRS (55.8% female, mean age: 69.28 years) were included in the subsequent analysis.

Table 1 presents the baseline characteristics of the participants included in the study. In the CHARLS cohort, 66.6% of participants were in the robust group, 30.2% were in the pre-frail group, and 3.2% were in the frail group; in the ELSA cohort, 83.2% were in the robust group, 13.4% in the pre-frail group, and 3.4% in the frail group; in the HRS cohort, 59.2% were in the robust group, 30.3% in the pre-frail group, and 10.5% in the frail group. At baseline across the three cohorts, females had a higher rate of pre-frailty and frailty compared to males, and individuals in the pre-frail and frail groups were older. In the ELSA and HRS cohorts, ever-smokers had higher rates of pre-frailty and frailty, while in CHARLS, non-smokers showed a higher rate. Additionally, participants who were unmarried or in other marital statuses, had lower education levels, poorer sleep quality, did not consume alcohol, and had hypertension or diabetes were more likely to be in the pre-frail and frail groups.

**Table 1:** Baseline characteristics of included participants.

Variables	CHARLS (*n* = 10,624)				ELSA (*n* = 4945)				HRS (*n* = 11,439)			
	Robust	Pre-frail	Frail	P value	Robust	Pre-frail	Frail	P value	Robust	Pre-frail	Frail	P value
Number (%)	6731 (66.6)	3053 (30.2)	322 (3.2)		4114 (83.2)	663 (13.4)	168 (3.4)		6769 (59.2)	3469 (30.3)	1201 (10.5)	
Age, mean (SD)	56.83 (8.62)	60.52 (9.80)	64.92 (10.24)	<0.001	64.97 (8.50)	70.79 (10.80)	74.40 (11.35)	<0.001	64.25 (9.33)	69.96 (10.70)	73.63 (11.36)	<0.001
Sex, n (%)												
Male	3867 (68.7)	1439 (25.6)	322 (5.7)	<0.001	2023 (85.5)	264 (11.2)	78 (3.3)	0.02	3105 (61.4)	1518 (30.0)	434 (8.6)	<0.001
Female	2859 (57.3)	1612 (32.3)	517 (10.4)		2091 (81.0)	399 (15.5)	90 (3.5)		3663 (57.4)	1951 (30.6)	767 (12.0)	
Marital status, n (%)												
Married or partnered	6212 (65.5)	2615 (27.6)	653 (6.9)	<0.001	2974 (87.9)	337 (10.0)	72 (2.1)	<0.001	4596 (65.9)	1907 (27.3)	474 (6.8)	<0.001
Other marital status	519 (45.4)	438 (38.3)	187 (16.3)		1139 (73.0)	326 (20.9)	96 (6.1)		2154 (48.6)	1553 (35.0)	725 (16.4)	
Education, n (%)												
Below high school	5435 (60.2)	2788 (30.9)	799 (8.9)	<0.001	2219 (78.4)	479 (16.9)	134 (4.7)	<0.001	792 (38.1)	847 (40.8)	438 (21.1)	<0.001
High school	797 (82.6)	142 (14.7)	26 (2.7)		761 (86.6)	98 (11.1)	20 (2.3)		1906 (54.8)	1176 (33.8)	393 (11.3)	
College or above	489 (78.4)	121 (19.4)	14 (2.2)		1080 (92.5)	74 (6.3)	13 (1.1)		4040 (69.2)	1433 (24.6)	363 (6.2)	
Sleeping status, n (%)												
good	3962 (70.9)	1400 (25.0)	230 (4.1)	<0.001	3203 (86.8)	395 (10.7)	92 (2.5)	<0.001	4737 (65.6)	1977 (27.4)	510 (7.1)	<0.001
poor	2079 (49.7)	1546 (36.9)	561 (13.4)		897 (72.4)	268 (21.6)	74 (6.0)		2009 (48.4)	1470 (35.4)	675 (16.2)	
Smoking status, n (%)												
Never smokers	3769 (61.0)	1836 (29.7)	569 (9.2)	<0.001	3469 (83.8)	539 (13.0)	132 (3.2)	<0.001	3329 (62.1)	1507 (28.1)	521 (9.7)	<0.001
Ever smokers	2959 (66.5)	1217 (27.4)	271 (6.1)		645 (80.1)	124 (15.4)	36 (4.5)		3343 (56.1)	1941 (32.6)	677 (11.4)	
Drinking status, n (%)												
Never drinkers	4028 (59.3)	2100 (30.9)	665 (9.8)	<0.001	310 (70.1)	93 (21.0)	39 (8.8)	<0.001	2365 (49.3)	1683 (35.1)	753 (15.7)	<0.001
Ever drinkers	2695 (70.5)	953 (24.9)	175 (4.6)		3476 (86.1)	470 (11.6)	89 (2.2)		4401 (66.3)	1786 (26.9)	448 (6.8)	
Hypertension, n (%)												
No	5493 (66.6)	2210 (26.8)	541 (6.6)	<0.001	3877 (83.3)	616 (13.2)	160 (3.4)	0.226	3430 (71.0)	1114 (23.1)	285 (5.9)	<0.001
Yes	1211 (52.1)	826 (35.5)	289 (12.4)		237 (81.2)	47 (16.1)	8 (2.7)		3232 (50.3)	2296 (35.7)	897 (14.0)	
Diabetes, n (%)												
No	6437 (64.2)	2831 (28.3)	751 (7.5)	<0.001	4040 (83.4)	645 (13.3)	161 (3.3)	0.198	5659 (63.1)	2546 (28.4)	760 (8.5)	<0.001
Yes	244 (47.4)	191 (37.1)	80 (15.5)		74 (74.7)	18 (18.2)	7 (7.1)		1063 (44.9)	884 (37.3)	422 (17.8)	

### 3.2 Longitudinal association between frailty and pain

In this study, data from the longitudinal databases CHARLS (China), ELSA (UK), and HRS(US) were utilized, and generalized linear mixed-effects models were applied to analyze the association between frailty status/frailty index and pain. Table 2 presents the results of the generalized linear mixed-effects models, which illustrate the longitudinal association between frailty status/frailty index and pain. The results indicate that in the CHARLS, ELSA, and HRS databases, both pre-frail and frail states were found to significantly increase the likelihood of pain compared to a robust state, with an increase in pain likelihood observed as the frailty index rose. Specifically, in the unadjusted models (Model 1) across the three cohorts, pre-frail individuals were 3.95 times (CHARLS, OR 3.95, 95% CI 3.70–4.22), 4.26 times (ELSA, OR 4.26, 95% CI 3.76–4.82), and 3.82 times (HRS, OR 3.82, 95% CI 3.51–4.17) more likely to experience pain compared to those in a robust state. Frail individuals had even higher odds, at 9.51 times (CHARLS, OR 9.51, 95% CI 8.62–10.49), 9.8 times (ELSA, OR 9.80, 95% CI 8.08–11.89), and 11.15 times (HRS, OR 11.15, 95% CI 9.98–12.45) compared to robust individuals. Furthermore, with each 0.1 increase in the frailty index, the likelihood of pain increased by 1.32 times (CHARLS, OR 2.32, 95% CI 2.25–2.40), 1.38 times (ELSA, OR 2.38, 95% CI 2.24–2.52), and 1.1 times (HRS, OR 2.10, 95% CI 2.03–2.17).

**Table 2:** Longitudinal association between frailty and pain.

		Model 1 OR (95% CI)	Model 2 OR (95% CI)	Model 3 OR (95% CI)	Model 4 OR (95% CI)
CHARLS					
	robust	1.00	1.00	1.00	1.00
	Pre-frail	3.95 (3.70, 4.22)	3.82 (3.57, 4.08)	3.60 (3.36, 3.86)	3.82 (3.51, 4.15)
	Frail	9.51 (8.62, 10.49)	9.12 (8.24, 10.10)	8.93 (8.00, 9.95)	10.44 (9.05, 12.05)
	Frailty index (per 0.1 unit)	2.32 (2.25, 2.40)	2.30 (2.23, 2.38)	2.35 (2.26, 2.44)	2.56 (2.44, 2.69)
ELSA					
	robust	1.00	1.00	1.00	1.00
	Pre-frail	4.26 (3.76, 4.82)	4.38 (3.85, 4.98)	3.708 (3.707, 3.709)	4.29 (3.74, 4.93)
	Frail	9.80 (8.08, 11.89)	10.65 (8.67, 13.07)	8.01 (8.00, 8.02)	10.14 (8.05, 12.76)
	Frailty index (per 0.1 unit)	2.38 (2.24, 2.52)	2.49 (2.33, 2.65)	2.54 (2.36, 2.73)	2.55 (2.37, 2.74)
HRS					
	robust	1.00	1.00	1.00	1.00
	Pre-frail	3.82 (3.51, 4.17)	3.758 (3.757, 3.759)	4.27 (3.90, 4.67)	4.17 (3.81, 4.57)
	Frail	11.15 (9.98, 12.45)	9.901 (9.898, 9.903)	13.53 (11.97, 15.29)	13.27 (11.71, 15.03)
	Frailty index (per 0.1 unit)	2.10 (2.03, 2.17)	2.34 (2.25, 2.43)	2.29 (2.20, 2.38)	2.28 (2.19, 2.37)

After gradually adjusting for demographic characteristics (gender, age, marital status, education level), lifestyle factors (smoking, alcohol consumption, sleep quality), and chronic conditions (hypertension, diabetes) in Models 2 through 4, the association between frailty and pain weakened but remained significant. In the fully adjusted model (Model 4), pre-frail individuals had 3.82 times (CHARLS, OR 3.82, 95% CI 3.51–4.15), 4.29 times (ELSA, OR 4.29, 95% CI 3.74–4.93), and 4.17 times (HRS, OR 4.17, 95% CI 3.81–4.57) the likelihood of experiencing pain compared to robust individuals, while frail individuals in the three cohorts were 10.44 times (CHARLS, OR 10.44, 95% CI 9.05–12.05), 10.14 times (ELSA, OR 10.14, 95% CI 8.05–12.76), and 13.27 times (HRS, OR 13.27, 95% CI 11.71–15.03) more likely to experience pain. For each 0.1 increase in the frailty index, the likelihood of pain increased by 1.56 times (CHARLS, OR 2.56, 95% CI 2.44–2.69), 1.55 times (ELSA, OR 2.55, 95% CI 2.37–2.74), and 1.28 times (HRS, OR 2.28, 95% CI 2.19–2.37) across the three cohorts.

The findings from all three databases consistently show that as frailty status worsens, the likelihood of experiencing pain significantly increases. Analyses across different databases and countries indicate that frailty consistently affects pain across diverse populations, with particularly strong effects observed in Western populations. Even after accounting for various factors, frailty remains a strong predictor of pain occurrence.

### 3.3 Longitudinal association between frailty and pain degrees

Data from ELSA and HRS were utilized to analyze the association between frailty status/frailty index and pain degrees by fitting Cumulative Link Mixed Models (CLMMs). Table 3 presents the CLMMs' results, which illustrate the longitudinal association between frailty status/frailty index and pain degrees. In both databases, individuals in the pre-frail and frail states reported significantly higher degrees of pain compared to robust individuals, with the risk increasing as the degree of frailty worsened. In the unadjusted models (Model 1), the risk of experiencing higher pain degrees for pre-frail individuals was 2.20 times that of robust individuals (ELSA, OR 2.20, 95% CI 1.78–2.71) and 2.00 times (HRS, OR 2.00, 95% CI 1.69–2.35). For individuals in the frail state, the risk was significantly higher, at 5.27 times (ELSA, OR 5.27, 95% CI 3.99–6.96) and 4.81 times (HRS, OR 4.81, 95% CI 3.99–5.80) compared to the robust individuals. Additionally, for every 0.1-unit increase in the frailty index, the risk of experiencing higher pain degrees increased by 74% (ELSA, OR 1.74, 95% CI 1.59–1.90) and by 58% (HRS, OR 1.58, 95% CI 1.50–1.69).

**Table 3:** Longitudinal association between frailty and pain degrees.

		Model 1 OR (95% CI)	Model 2 OR (95% CI)	Model 3 OR (95% CI)	Model 4 OR (95% CI)
ELSA					
	robust	1.00	1.00	1.00	1.00
	Pre-frail	2.20 (1.78, 2071)	2.03 (1.64, 2.51)	2.00 (1.59, 2.51)	2.03 (1.62, 2.56)
	Frail	5.27 (3.99, 6.96)	4.41 (3.29, 5.92)	4.44 (3.21, 6.15)	4.72 (3.39, 6.56)
	Frailty index (per 0.1 unit)	1.74 (1.59, 1.90)	1.63 (1.49, 1.79)	1.64 (1.48, 1.82)	1.69 (1.52, 1.87)
HRS					
	robust	1.00	1.00	1.00	1.00
	Pre-frail	2.00 (1.69, 2.35)	1.98 (1.68, 2.34)	1.91 (1.61, 2.26)	2.00 (1.69, 2.37)
	Frail	4.81 (3.99, 5.80)	4.66 (3.84, 5.66)	4.42 (3.62, 5.40)	4.47 (3.65, 5.46)
	Frailty index (per 0.1 unit)	1.58 (1.50, 1.69)	1.60 (1.52, 1.69)	1.57 (1.48, 1.66)	1.55 (1.47, 1.64)

Cumulative Link Mixed Models (CLMMs) were fitted to analyze the association between frailty and pain degrees. Pain degrees (mild, moderate, severe) were used as the outcome variable, with mild as the control.

OR (Odds Ratio) indicates the direction and intensity of the effect of frailty on the occurrence of higher pain degrees.

Model 1 was unadjusted; Model 2 adjusted for gender, age, marital status, and education level; Model 3 further adjusted for smoking status, drinking status, and sleep quality based on Model 2; and Model 4 further adjusted for hypertension and diabetes based on Model 3.

**Table 4:** Longitudinal association between frailty and pain areas.

		Body pain areas			
		Head & neck	Trunk	Limbs	Oral
CHARLS					
robust		1.00	1.00	1.00	No data available
Pre-frail		3.34 (3.03, 3.68)	3.68 (3.37, 4.03)	3.51 (3.23, 3.82)	No data available
Frail		7.86 (6.90, 8.95)	8.78 (7.68, 10.03)	10.47 (9.20, 11.90)	No data available
Frailty index (per 0.1 unit)		2.03 (1.95, 2.12)	2.20 (2.11, 2.30)	2.41 (2.31, 2.52)	No data available
ELSA					
robust		No data available	1.00	1.00	1.00
Pre-frail		No data available	4.59 (4.01, 5.25)	4.84 (4.20, 5.57)	3.22 (2.08, 4.98)
Frail		No data available	10.13 (8.40, 12.22)	12.98 (10.72, 15.72)	10.51 (6.77, 16.33)
Frailty index (per 0.1 unit)		No data available	2.16 (2.04, 2.30)	2.24 (2.12, 2.38)	1.89 (1.70, 2.10)

All results are after adjusting for covariates: gender, age, marital status, education level, smoking status, drinking status, sleep quality, hypertension, and diabetes.

After progressively adjusting for demographic factors (Model 2), lifestyle factors (Model 3), and health conditions (Model 4), the risks for both pre-frail and frail states were slightly reduced but remained significant. In the final model (Model 4), the odds ratio (OR) for pre-frail individuals was 2.03 (ELSA, 95% CI 1.62–2.56) and 2.00 (HRS, 95% CI 1.69–2.37), while for frail individuals, the OR was 4.72 (ELSA, 95% CI 3.39–6.56) and 4.47 (HRS, 95% CI 3.65–5.46). The impact of the frailty index was also slightly attenuated but remained significant in the final model, with ORs of 1.69 (ELSA, 95% CI 1.52–1.87) and 1.55 (HRS, 95% CI 1.47–1.64).

Although the OR values varied slightly between the ELSA and HRS databases across the models, the overall trends were consistent. Both the pre-frail and frail states were significantly associated with higher degrees of pain in both databases, with the effect being more pronounced in the frail state.

### 3.4 Longitudinal association between frailty and pain areas

Besides, the association between frailty status/frailty index and pain areas was analyzed using data from CHARLS and ELSA. Table 4 presents the results of the generalized linear mixed-effects models. In the CHARLS database, frailty status and index were significantly associated with pain in the head and neck, trunk, and limbs, with a stronger impact observed in the frail state. Compared to robust individuals, pre-frail individuals had a 3.34-fold increased risk of experiencing head and neck pain (OR 3.34, 95% CI 3.03–3.68), while frail individuals had a 7.86-fold increased risk (OR 7.86, 95% CI 6.90–8.95). For trunk pain, the risk was 3.68 times higher in pre-frail individuals (OR 3.68, 95% CI 3.37–4.03) and increased to 8.78 times higher in frail individuals (OR 8.78, 95% CI 7.68–10.03). For limb pain, pre-frail individuals had a 3.51-fold increased risk (OR 3.51, 95% CI 3.23–3.82), and the risk rose sharply to 10.47 times in frail individuals (OR 10.47, 95% CI 9.20–11.90). For every 0.1-unit increase in the frailty index, the risk of head and neck pain increased 1.03-fold (OR 2.03, 95% CI 1.95–2.12), trunk pain increased 1.2-fold (OR 2.20, 95% CI 2.11–2.30), and limbs pain increased 1.41-fold (OR 2.41, 95% CI 2.31–2.52).

In the ELSA database, frailty status and index were also significantly associated with pain in the trunk, limbs, and oral. Pre-frail individuals had a 4.59-fold increased risk of experiencing trunk pain (OR 4.59, 95% CI 4.01–5.25), while frail individuals had a 10.13-fold increased risk (OR 10.13, 95% CI 8.40–12.22). For limb pain, the risk was 4.84 times higher in pre-frail individuals (OR 4.84, 95% CI 4.20–5.57) and 12.98 times higher in frail individuals (OR 12.98, 95% CI 10.72–15.72). Regarding oral pain, pre-frail individuals had a 3.22-fold increased risk (OR 3.22, 95% CI 2.08–4.98), and frail individuals had a 10.51-fold increased risk (OR 10.51, 95% CI 6.77–16.33). For every 0.1-unit increase in the frailty index, the risk of trunk pain increased 1.16-fold (OR 2.16, 95% CI 2.04–2.30), limb pain increased 1.24-fold (OR 2.24, 95% CI 2.12–2.38), and oral pain increased by 89% (OR 1.89, 95% CI 1.70–2.10).

Overall, the CHARLS database showed significant associations with head and neck pain. In contrast, the ELSA database demonstrated a significant association between frailty and oral pain. Both databases consistently showed significant risks for trunk and limb pain, with frail individuals exhibiting the highest risks (ranging from 8 to 13 times). The frailty index was a key predictor of pain in both databases, although its impact varied across body regions: in the CHARLS database, the frailty index had the strongest effect on limb pain (OR = 2.41), while in the ELSA database, it had the strongest effect on trunk pain (OR = 2.16).

### 3.5 Sensitivity analysis

The sensitivity analysis follows the same trend as the results of the main analysis. The results of the COX model are detailed in Supplementary Table S3. This reinforces the reliability and consistency of the study's results.

## 4 Discussion

This study, utilizing data from the three major databases CHARLS (China), ELSA (UK), and HRS (US), examined the longitudinal associations between frailty and the occurrence of pain, the degrees of pain, and the areas of pain, while also analyzing the impact of covariates such as gender, age, marital status, education level, sleep quality, smoking, alcohol consumption, hypertension, and diabetes. The results demonstrate that frailty significantly impacts the risk of pain, the degrees of pain and the areas of pain. And this association is consistently observed across older populations in different countries.

Given the rapid growth of the global aging population, frailty has become a common health issue. Previous studies have shown a close association between frailty and pain. Blyth et al. first revealed that individuals with frailty (≥3 frailty phenotype criteria) are more likely to report pain [31]. Furthermore, frailty is an independent predictor of both acute and chronic postoperative pain (APSP and CPSP) following total knee arthroplasty (TKA) in older patients [32], and frail individuals are at higher risk of chronic pain following cardiac surgery [33]. Similarly, among middle-aged and older adults in China, a U-shaped relationship was observed between FI and the prevalence of low back pain (LBP), indicating that both extremely low and high frailty levels are associated with an increased risk of LBP [34]. Another study confirmed a significant association between frailty and LBP pain intensity, showing that frail older women experience higher pain intensity compared to robust individuals, suggesting a potential pathophysiological link between frailty and pain exacerbation [35]. In hospitalized cancer patients, FI has also been shown to correlate with persistent pain and its intensity [36]. These findings are consistent with our study's conclusion that frailty status significantly increases the risk of pain. Furthermore, frailty is associated with more severe pain trajectories; frail individuals are 5–6 times more likely than robust individuals to experience “severe” or “very severe” pain trajectories over a nine-year follow-up, suggesting that fatigue and slow gait may be key drivers [37]. Additional longitudinal studies have confirmed the bidirectional relationship between frailty and pain [38]. The literature above strongly supports our study and provides evidence that frailty status is an important predictor of pain risk across different countries and populations.

The mechanism through which frailty affects pain is complex, involving multiple physiological and psychological pathways. First, frailty is often accompanied by a decline in muscle strength and mass, impacting individuals' ability to engage in activities in various environments and limiting daily functioning, which may increase the risk of LBP and related injuries [39]. Additionally, the physical activity capacity of frail individuals is usually impaired, when regular physical activity and exercise reduces excitability of central neurons, measured by phosphorylation of the NR1 subunit of the NMDA receptor, alters neuroimmune signaling in the central nervous system, and increases release of endogenous opioids and serotonin in the brainstem pain inhibitory pathways [40]. Physical exercise has been shown to moderate the relationship between frailty and pain intensity, with higher levels of physical activity alleviating the negative impact of frailty on pain, thereby relieving pain symptoms [41]. The decline in central pain pathway function is another important mechanism underlying pain exacerbation in frail individuals. Frailty is often accompanied by degenerative changes in the central nervous system, making it more difficult for older adults to regulate pain perception and effectively cope with new nociceptive stimulus. Emotional disturbances, particularly depressive symptoms, may be associated with changes in the brain that affect the descending pain inhibitory system, potentially increasing the association between frailty and pain. Furthermore, frailty is associated with dysregulation of the hypothalamic-pituitary-adrenal (HPA) axis, which is considered an important risk factor for chronic widespread pain [31]. These central mechanisms may lead to decreased pain tolerance and intensified pain symptoms in frail individuals. The relationship between frailty and pain also involves changes in the immune and inflammatory systems. Studies have shown that frail individuals exhibit elevated levels of inflammatory markers such as TNF-α, IL-6, and C-reactive protein, and these pro-inflammatory mediators may activate nociceptors and cause pain. Additionally, with aging, inflammatory mediators may increase even without obvious disease, and this chronic low-grade inflammation may heighten pain sensitivity in frail individuals, resulting in more severe pain symptoms [35]. Frailty may also lead to muscle atrophy and joint instability, increasing sensitivity to biomechanical injury and exacerbating pain symptoms [42].

The results of this study indicate a significant gender difference in the occurrence of pain, with men showing a significantly lower incidence of pain compared to women. This finding further supports previous literature suggesting that women are more likely to report higher pain intensity and lower pain tolerance. Most of the patients in the study were women, who exhibited higher pain intensity in postoperative pain [43,44]. Biological mechanisms suggest that testosterone may provide a protective effect for men, while estrogen fluctuations might lower pain thresholds in women. As a result, women are more susceptible to pain-related conditions, such as headaches and musculoskeletal pain [45]. These differences further underscore the need for public health policies to focus on pain management in women, particularly middle-aged and older women, to improve their quality of life.

Besides, we found that the impact of age on pain occurrence varies across countries. In the samples from China and the United States, the risk of pain slightly decreased with increasing age, whereas no significant association was observed in the UK. This aligns with findings in global literature, which suggest that the prevalence of neck pain typically peaks in middle age and then declines with further aging [46]. Aging is a major risk factor for most chronic pain conditions, as the pain modulation system in older adults gradually weakens, leading to reduced pain sensitivity [47]. Research indicates that with aging, older adults exhibit reduced endogenous inhibition of pain sensitivity, a lower temporal summation threshold for nociceptive stimuli, and decreased levels of β-endorphins and GABA synthesis [47]. Therefore, understanding the complex effects of age on pain, as well as the changes in pain modulation systems associated with aging, is crucial for developing effective pain management strategies for the older population.

Our results also indicate that marital status is significantly associated with the occurrence of pain, particularly in the UK and the US, where individuals who are married or have partners face a higher risk of pain, whereas no significant effect was observed in China. This finding is consistent with previous studies, which suggest that married patients often experience greater life stress, especially married women who must balance work and family responsibilities, potentially exacerbating pain symptoms [48]. Furthermore, married individuals are more likely to suffer from high-disability chronic pain compared to those who are single, divorced, or widowed [49]. Research suggests that factors such as family conflict, inadequate coping mechanisms, and a lack of time for self-care may contribute to the worsening of headaches and chronic pain in married patients [48]. Therefore, this study underscores the importance of considering the impact of marital status on pain management, particularly for married patients in high-stress family environments.

Furthermore, this study found that educational attainment has a significant impact on the occurrence of pain. In China and the UK, individuals with higher education levels showed a lower incidence of pain, whereas in the US, individuals with higher education levels demonstrated an increased risk of pain. This aligns with findings from the 2010–2017 NHIS survey, which showed that individuals with lower education levels are at a higher risk of pain, particularly high school dropouts, who face a 30% higher risk of pain compared to high school graduates, while college graduates experience a 30% lower risk of pain. Education influences pain risk by improving individuals' economic resources and health behaviors [50]. However, educated but unemployed individuals may experience more pain due to mental health issues, such as chronic tension-type headaches [48]. Unemployment may offset the health advantages conferred by education, particularly within educated populations, highlighting the complex interplay of socioeconomic and psychological factors in pain occurrence.

Our study also found that poor sleep quality significantly increases the risk of pain occurrence. In the samples from China, the UK, and the US, poor sleep quality was closely associated with a higher risk of pain. This finding aligns with existing literature, which suggests a bidirectional relationship between poor sleep quality and chronic pain conditions such as neck and back pain [51]. Genetic studies have also shown that part of the genetic effects on neck and back pain are shared with sleep quality, while environmental factors play a smaller role in the association between pain and sleep [52]. Furthermore, insufficient sleep (<7 h) is a major predictor of physical pain reported by middle-aged and older adults [53]. Chronic pain patients often suffer from insomnia, where reduced sleep quality sensitizes the nervous system, making pain harder to tolerate, which in turn worsens sleep, creating a vicious cycle [54]. Therefore, in pain management, focusing on improving sleep quality may be an effective intervention to alleviate pain and enhance quality of life.

In addition, our results indicate that moderate high blood pressure is significantly associated with an increased risk of pain occurrence across the samples from the three countries. Hypertensive patients reported a significantly higher risk of pain compared to non-hypertensive individuals. This finding is consistent with existing literature, which indicates that older adults with hypertension are more likely to experience activity-limiting pain and report pain in multiple locations [47]. Hypertension may increase the prevalence of pain through various mechanisms, including heightened central pain sensitivity and reduced baroreceptor sensitivity, both of which weaken pain modulation functions [55]. Therefore, this study underscores the importance of pain management in hypertensive patients, particularly among older populations, where the comorbidity of hypertension and pain should be carefully addressed.

The findings of this study suggest that pain management should integrate multifaceted strategies, especially for the older adults. First, pain assessment must be emphasized. The PENS (Pain, Expectations/Emotions, Nutrition, Sleep) acronym (developed by Mark Greenwood) is one approach to guide pain assessment in frail older adults. PENS was originally developed as a patient-provider communication tool to manage pain and suffering, improve transparent and effective communication between patients, healthcare providers, and caregivers, and target pain-related factors particularly relevant for vulnerable older adults [56]. Besides, timely reassessment and evaluation is essential to monitor for improvement or deterioration in pain or function, adverse effects, and to optimize pain management. Our study highlights the significant impact of frailty on pain risk suggesting that interventions targeting frailty may help alleviate pain. Hence, frailty should be assessed in all older adults with pain using a screening tool like the EASY-Care Two-step Older persons Screening (EASY-Care TOS). Review patients’ medical history to identify potentially painful conditions and recognize that older adults may display other signs related to pain, including fatigue, inability to sleep, loss of appetite, and delirium [56]. Moreover, improving sleep quality and managing mental health [57] are essential for breaking the negative cycle of pain and frailty. In summary, routine pain assessment and management should be conducted for frail individuals, with early screening and intervention for psychological issues such as depression, and promotion of healthy lifestyle habits to effectively reduce the high comorbidity of pain and frailty. Future public health strategies and clinical practices should emphasize the dual management of pain and frailty to improve the quality of life for older patients.

This study has several strengths. To our knowledge, it is one of the few longitudinal studies investigating the relationship between frailty and pain risk, and the use of generalized linear mixed-effects models allows for optimal utilization of each year's follow-up data. Furthermore, this study includes three prospective cohorts from different ethnicities, with a rigorous study design and large sample size, and our findings are consistent across all three cohorts, indicating that our conclusions are generalizable. However, there are some limitations. First, the frailty score was calculated using self-reported data when many of the conditions could be unknown to the individual. Second, while we evaluated several variables concerning pain, the variables were limited to those consistently available across the three databases or in each follow-up year. Factors previously shown to be related to pain, such as physical activity (which had high levels of missing data in the annual follow-up of CHARLS) [58], could not be analyzed. Therefore, further research is needed to determine the association between study factors and pain changes after controlling for these unassessed variables.

## 5 Conclusion

This study, data from three prospective cohorts (CHARLS, ELSA, and HRS) were utilized to thoroughly examine the longitudinal association between frailty and pain. The results indicate that frailty significantly impacts the risk of pain, the degrees of pain and the areas of pain. And this association is consistently observed across older populations in different countries.

## Declaration of interests

The authors declare that they have no known competing financial interests or personal relationships that could have appeared to influence the work reported in this paper.
