# Supplementary material for: Longitudinal association between frailty and pain in three prospective cohorts of older population
Source: J Nutr Health Aging. 2025 Mar 23;29(6):100537. doi: 10.1016/j.jnha.2025.100537 (PMC12172954; doi:10.1016/j.jnha.2025.100537)
Supplement: Supplementary file 1 [file mmc1.docx]

| **Table S1: The 32 items used to construct the frailty index** | | | | |
| --- | --- | --- | --- | --- |
| No |  | | | Cut-off value |
|  | CHARLS | ELSA | HRS |  |
| 1 | Self-reported physician diagnosed heart disease | | | Yes = 1, No = 0 |
| 2 | Self-reported physician diagnosed stroke | | | Yes = 1, No = 0 |
| 3 | Self-reported physician diagnosed cancer | | | Yes = 1, No = 0 |
| 4 | Self-reported physician diagnosed arthritis | | | Yes = 1, No = 0 |
| 5 | Self-reported physician diagnosed chronic lung disease | | | Yes = 1, No = 0 |
| 6 | Self-reported physician diagnosed any emotional, nervous, or psychiatric problems | | | Yes = 1, No = 0 |
| 7 | Self-reported physician diagnosed memory-related disease | | | Yes = 1, No = 0 |
| 8 | Self-reported vision problems | Self-reported eyesight (while using lenses if appropriate) | | Yes = 1, No = 0 in the CHARLS; Poor or fair = 1, excellent, very good, or good = 0 in the ELSA and HRS |
| 9 | Self-reported hearing problems | Self-reported hearing (while using hearing aid if appropriate) | |  |
| 10 | Self-reported general health status | | | Very poor or poor = 1,  Very good, good, or fair = 0 |
| 11 | Difficulty with dressing | | | Yes = 1, No = 0 |
| 12 | Difficulty with bathing or showering | | | Yes = 1, No = 0 |
| 13 | Difficulty with eating | | | Yes = 1, No = 0 |
| 14 | Difficulty with getting in and out of bed | | | Yes = 1, No = 0 |
| 15 | Difficulty with using the toilet | | | Yes = 1, No = 0 |
| 16 | Difficulty with managing money | | | Yes = 1, No = 0 |
| 17 | Difficulty with taking medication | | | Yes = 1, No = 0 |
| 18 | Difficulty with shopping for groceries | | | Yes = 1, No = 0 |
| 19 | Difficulty with preparing meals | | | Yes = 1, No = 0 |
| 20 | Mobility: difficulty with walking 100 yards or one block | | | Yes = 1, No = 0 |
| 21 | Mobility: difficulty with getting up from a chair after sitting for long periods | | | Yes = 1, No = 0 |
| 22 | Mobility: difficulty with climbing several flights of stairs without resting | | | Yes = 1, No = 0 |
| 23 | Mobility: difficulty with lifting or carrying weights over 10 pounds/jins | | | Yes = 1, No = 0 |
| 24 | Mobility: difficulty with picking up a coin from the table | | | Yes = 1, No = 0 |
| 25 | Mobility: difficulty with stooping, kneeling, or crouching | | | Yes = 1, No = 0 |
| 26 | Mobility: difficulty with reaching arms above shoulder level | | | Yes = 1, No = 0 |
| 27 | Depression: feel depressed | | | Most/Occasionally = 1, Little/Rarely = 0 in the CHARLS; Yes = 1, No = 0 in the ELSA and HRS |
| 28 | Depression: feel everything is an effort | | |  |
| 29 | Depression: feel lonely | | |  |
| 30 | Depression: have trouble getting going | | |  |
| 31 | Depression: feel happy | | | Most/Occasionally = 0, Little/Rarely = 1 in the CHARLS; Yes = 0, No = 1 in the ELSA and HRS |
| 32 | Cognition: (memory test score + orientation test score) **/** 14 | | | Continuous variable, ranging  from 0 to 1 |
| Memory-related disease included Alzheimer’s disease, dementia.  The memory test score was the average of words which were not recalled in the immediate and delayed word recall tasks. The memory score ranged from 0 to 10. The orientation test comprised 4 questions about the day of the week, the month, the date of the month, and the year. One point was given for each wrong answer, and the range was from 0 to 4. [1] [1] Di He, Zhaoping Wang, Jun Li, Kaixin Yu, Yusa He, Xinyue He, et al. Changes in frailty and incident cardiovascular disease in three prospective cohorts. European Heart journal. 2024, 45:1058-1068. | | | | |
