# Supplementary material for: Longitudinal association between frailty and pain in three prospective cohorts of older population
Source: J Nutr Health Aging. 2025 Mar 23;29(6):100537. doi: 10.1016/j.jnha.2025.100537 (PMC12172954; doi:10.1016/j.jnha.2025.100537)
Supplement: Supplementary file 2 [file mmc2.docx]

| **Table S2:** Baseline characteristics of included and excluded participants | | | | | | | | |
| --- | --- | --- | --- | --- | --- | --- | --- | --- |
| Variables | CHARLS | |  | ELSA | |  | HRS | |
|  | Excluded | Included |  | Excluded | Included |  | Excluded | Included |
| Number (%) | 6995 (39.7) | 10624 (60.3) |  | 5587 (53.0) | 4945 (47.0) |  | 9071 (44.2) | 11439 (55.8) |
| Age, mean (SD), years | 59.85 (11.09) | 58.53 (9.44) |  | 67.16 (11.55) | 66.07 (9.30) |  | 66.61 (12.79) | 66.97 (10.56) |
| Sex, n (%) |  |  |  |  |  |  |  |  |
| Male | 2811 (40.2) | 5628 (53.0) |  | 2341 (41.9) | 2365 (47.8) |  | 3476 (38.3) | 5057 (44.2) |
| Female | 4179 (59.8) | 4988 (47.0) |  | 3246 (58.1) | 2580 (52.2) |  | 5595 (61.7) | 6381 (55.8) |
| Marital status, n (%) |  |  |  |  |  |  |  |  |
| Married or partnered | 5909 (84.8) | 9480 (89.2) |  | 3697 (66.2) | 3383 (68.4) |  | 4971 (55.0) | 6977 (61.2) |
| Other marital status | 1059 (15.2) | 1144 (10.8) |  | 1888 (33.8) | 1561 (31.6) |  | 4074 (45.0) | 4432 (38.8) |
| Education, n (%) |  |  |  |  |  |  |  |  |
| Below high school | 6315 (90.7) | 9022 (85.0) |  | 3816 (69.7) | 2832 (58.1) |  | 2295 (25.4) | 2077 (18.2) |
| High school | 413 (5.9) | 965 (9.1) |  | 836 (15.3) | 879 (18.0) |  | 2956 (32.8) | 3475 (30.5) |
| College or above | 232 (3.3) | 624 (5.9) |  | 820 (15.0) | 1167 (23.9) |  | 3773 (41.8) | 5836 (51.2) |
| Smoking status, n (%) |  |  |  |  |  |  |  |  |
| Never smokers | 4439 (64.2) | 6174 (58.1) |  | 4493 (80.5) | 4140 (83.7) |  | 3610 (40.3) | 5357 (47.3) |
| Ever smokers | 2475 (35.8) | 4447 (41.9) |  | 1089 (19.5) | 805 (16.3) |  | 5357 (59.7) | 5961 (52.7) |
| Drinking status, n (%) |  |  |  |  |  |  |  |  |
| Never drinkers | 4976 (72.0) | 6793 (64.0) |  | 735 (16.9) | 442 (9.9) |  | 4713 (52.0) | 4801 (42.0) |
| Ever drinkers | 1937 (28.0) | 3823 (36.0) |  | 3617 (83.1) | 4035 (90.1) |  | 4357 (48.0) | 6635 (58.0) |
| Sleeping status, n (%) |  |  |  |  |  |  |  |  |
| Good | 2198 (35.4) | 5592 (57.2) |  | 2800 (56.1) | 3690 (74.9) |  | 3655 (40.6) | 7224 (63.5) |
| Bad | 4008 (64.6) | 4186 (42.8) |  | 2189 (43.9) | 1239 (25.1) |  | 5350 (59.4) | 4154 (36.5) |
| Hypertension, n (%) |  |  |  |  |  |  |  |  |
| Yes | 4920 (71.6) | 8244 (78.0) |  | 5066 (90.8) | 4653 (94.1) |  | 2956 (33.1) | 4829 (42.9) |
| No | 1953 (28.4) | 2326 (22.0) |  | 511 (9.2) | 292 (5.9) |  | 5988 (66.9) | 6425 (57.1) |
| Diabetes, n (%) |  |  |  |  |  |  |  |  |
| Yes | 6369 (93.0) | 10019 (95.1) |  | 5371 (96.3) | 4846 (98.0) |  | 6409 (71.5) | 8965 (79.1) |
| No | 477(7.0) | 515 (4.9) |  | 206 (3.7) | 99 (2.0) |  | 2554 (28.5) | 2369 (20.9) |
| CHARLS, China Health and Retirement Longitudinal Study; ELSA, English Longitudinal Study of Ageing; HRS, Health and Retirement Study | | | | | | | | |
