# Supplementary material for: Longitudinal association between frailty and pain in three prospective cohorts of older population
Source: J Nutr Health Aging. 2025 Mar 23;29(6):100537. doi: 10.1016/j.jnha.2025.100537 (PMC12172954; doi:10.1016/j.jnha.2025.100537)
Supplement: Supplementary file 3 [file mmc3.docx]

| **Table S3: Longitudinal association between frailty and pain (results form COX regression model)** | | | | | | |
| --- | --- | --- | --- | --- | --- | --- |
|  |  | events/n (%) | Model 1 HR (95% CI) | Model 2 HR (95% CI) | Model 3 HR (95% CI) | Model 4 HR (95% CI) |
| CHARLS |  |  |  |  |  |  |
|  | robust | 2850/4165 (0.68) | 1.00 | 1.00 | 1.00 | 1.00 |
|  | Pre-frail | 1592/1896 (0.84) | 1.66 (1.56, 1.76) | 1.59 (1.50, 1.70) | 1.54 (1.45, 1.65) | 1.54 (1.45, 1.65) |
|  | Frail | 412/438 (0.94) | 2.68 (2.42, 2.98) | 2.53 (2.27, 2.82) | 2.38 (2.13, 2.66) | 2.38 (2.13, 2.65) |
| ELSA |  |  |  |  |  |  |
|  | robust | 1669/4304 (0.39) | 1.00 | 1.00 | 1.00 | 1.00 |
|  | Pre-frail | 1095/1847 (0.59) | 1.92 (1.62, 2.26) | 1.79 (1.51, 2.23) | 1.67 (1.40, 1.99) | 1.66 (1.40, 1.98) |
|  | Frail | 300/424 (0.71) | 2.14 (1.46, 3.14) | 2.01 (1.37, 2.95) | 1.81 (1.23, 2.67) | 1.81 (1.23, 2.67) |
| HRS |  |  |  |  |  |  |
|  | robust | 1048/2208 (0.47) | 1.00 | 1.00 | 1.00 | 1.00 |
|  | Pre-frail | 160/228 (0.70) | 1.86 (1.73, 2.01) | 1.94 (1.79, 2.10) | 1.89 (1.74, 2.05) | 1.87 (1.72, 2.03) |
|  | Frail | 27/36 (0.75) | 2.65 (2.34, 3.00) | 2.77 (2.43, 3.15) | 2.64 (2.31, 3.02) | 2.58 (2.26, 2.96) |
| Model 1 was unadjusted; Model 2 adjusted for gender, age, marital status, and education level;  Model 3 further adjusted for smoking status, drinking status, and sleep quality based on Model 2;  and Model 4 further adjusted for hypertension and diabetes based on Model 3. | | | | | | |
|  |  |  |  |  |  |  |
|  |  |  |  |  |  |  |
| CHARLS, China Health and Retirement Longitudinal Study; ELSA, English Longitudinal Study of Ageing; HRS, Health and Retirement Study | | | | | | |
