# Supplementary material for: Longitudinal association between frailty and pain in three prospective cohorts of older population
Source: J Nutr Health Aging. 2025 Mar 23;29(6):100537. doi: 10.1016/j.jnha.2025.100537 (PMC12172954; doi:10.1016/j.jnha.2025.100537)
Supplement: Supplementary file 4 [file mmc4.docx]

**Supplemental Methods**

**Study designs of** **the CHARLS, ELSA, and HRS**

The China Health and Retirement Longitudinal Study (CHARLS) was a prospective cohort study conducted in China. In wave 1, a nationally representative sample of 17,708 participants was recruited from 28 provinces in 2011 via multistage probability sampling. The primary aim of this study was to recruit participants aged ≥ 45 years, but some participants aged 40 to 44 years also attended the baseline survey. All 17,708 participants underwent face-to-face interviews by the trained staff using the standardized questionnaire to collect data on sociodemographic information, lifestyles, and health-related information. Among 17,708 participants, 13,978 participants conducted anthropometric measurements to collect data on height, weight, waist circumference, hip circumference, blood pressure, grip strength, and so on. In addition, 11,847 participants provided blood samples for the laboratory test. Biochemical indicators, including total cholesterol, high-density lipoprotein cholesterol, low-density lipoprotein cholesterol, glycated hemoglobin, fasting blood glucose, and C-reactive protein were measured by the blood test. The follow-up surveys were conducted in 2013 (wave 2), 2015 (wave 3), 2018 (wave 4), 2020(wave 5) with questionnaire interviews and anthropometric measurements. Blood samples were collected in 2015 again, and biochemical indicators were measured by the blood test.

The English Longitudinal Study of Ageing (ELSA) was a prospective cohort study conducted in the UK. In wave 1, a nationally representative sample of 11,391 participants aged ≥50 years was recruited in 2002-2003. For these participants, face-to-face interviews combined with self-completion questionnaires were conducted by the trained staff to collect data on sociodemographic information, lifestyles, and health-related information. Furthermore, additional nurse visits were conducted for the assessments of anthropometric (including height, weight, waist circumference, hip circumference, blood pressure, grip strength, and so on) and blood biochemical indicators (including total cholesterol, high-density lipoprotein cholesterol, low-density lipoprotein cholesterol, glycated hemoglobin, fasting blood glucose, and C-reactive protein). The first nurse visit began in wave 2 (2004-2005), which included 7,666 participants from the core members. Routine follow-up surveys were conducted every two years since wave 1, and nurse visits were conducted every four years since wave 2.

The Health and Retirement Study (HRS) was also a prospective and nationally representative cohort study conducted in the USA. For wave 1, HRS recruited participants aged 51 to 61 years in 1992 combined with the Asset and Health Dynamics of the Oldest Old Study which enrolled participants aged ≥70 years in 1993. In 1998, HRS also merged two studies (the Children of the Depression and the War Babies) to make the sample fully representative of the USA population aged ≥50 years. Since 1992, participants were interviewed every 2 years to collect data on sociodemographic information, lifestyles, and health-related information. Before 2004 (wave 7), most interviews were conducted by telephone, except for participants aged ≥80 who were offered face-to-face interviews. Since 2006 (wave 8), half of the participants performed enhanced face-to-face interviews (EFTF), while the other half carried out telephone interviews. In the EFTF interviews, physical tests were conducted to collect data on height, weight, waist circumference, blood pressure, grip strength, and so on. Meanwhile, blood samples were assayed for five biochemical indicators: total cholesterol, high-density lipoprotein cholesterol, glycated hemoglobin, C-reactive protein, and cystatin C. Routine follow-up surveys were performed every two years since wave 1.

**Reference**

1. Sato K, Noguchi H, Inoue K, Kawachi I, Kondo N. Retirement and cardiovascular disease: a longitudinal study in 35 countries. *Int J Epidemiol* 2023;**52**:1047-1059. doi: 10.1093/ije/dyad058

2. Gao K, Cao LF, Ma WZ, Gao YJ, Luo MS, Zhu J*, et al.* Association between sarcopenia and cardiovascular disease among middle-aged and older adults: Findings from the China health and retirement longitudinal study. *EClinicalMedicine* 2022;**44**:101264. doi: 10.1016/j.eclinm.2021.101264

3. Bu F, Steptoe A, Fancourt D. Relationship between loneliness, social isolation and modifiable risk factors for cardiovascular disease: a latent class analysis. *J Epidemiol Community Health* 2021;**75**:749-754. doi: 10.1136/jech-2020-215539

4. Skoblow HF, Proulx CM. C-Reactive Protein, Subjective Aging, and Incident Cardiovascular Disease: A Mediation Model. *J Gerontol B Psychol Sci Soc Sci* 2022;**77**:1654-1658. doi: 10.1093/geronb/gbac051

5. Xie W, Zheng F, Yan L, Zhong B. Cognitive Decline Before and After Incident Coronary Events. *J Am Coll Cardiol* 2019;**73**:3041-3050. doi: 10.1016/j.jacc.2019.04.019

6. Glymour MM, Avendano M. Can self-reported strokes be used to study stroke incidence and risk factors?: evidence from the health and retirement study. *Stroke* 2009;**40**:873-879. doi: 10.1161/strokeaha.108.529479
